# The effect of right ventricle septal pacing versus apical pacing in dual-chamber pacemakers on patients’ anxiety and depression: a one-year follow-up study

**DOI:** 10.1186/s43044-024-00513-2

**Published:** 2024-07-04

**Authors:** Hassan El-Shirbiny, Reda Biomy, Wael Anwar Haseeb, Islam Saboukh

**Affiliations:** Cardiology Department, Faculty of Medicine, Kafrelsheikh University, Kafr El Sheikh, 33155 Egypt

**Keywords:** Anxiety, Depression, Psychological effect, Pacemaker, Dual chamber, Pacing, R.V. lead

## Abstract

**Background:**

Anxiety and depression are potentially harmful outcomes of permanent cardiac pacemakers. Dual-chamber P.P.M. is frequently used to treat life threatening bradycardia. The study aims to estimate the effect of the right ventricular PM lead position on recipients’ anxiety and depression before, 6 months, and 1 year after implantation.

**Results:**

A statistically significant correlation was discovered between the studied groups regarding HADS depression score after 6 months (*p* 0.013) and 1 year (*p* 0.013). A statistically non-significant difference was found among the studied groups at any point of time regarding baseline (*p* 0.063), after 6 months (*p* 0.054), or after 1 year (*p* 0.099). Significance was found between HADS anxiety score (*p* 0.015) or depression score after 1 year and the incidence of complications (*p* 0.001).

**Conclusions:**

A strong relationship was found between the level of depression and the R.V. site of implantation, as patients with the apical group had higher levels of depression post-implantation. The septal position has less stress and depression on the patient’s well-being than the apical one.

## Background

The impact of R.V pacing and the placement of the pacemaker lead on ventricular dyssynchrony is widely recognized. This condition mimics the symptoms of LBBB and leads to inadequate electrical and mechanical stimulation of the ventricles. These effects have been observed in various experimental and clinical studies [1]. R.V pacing alters cardiac perfusion, hemodynamics, metabolism, and mechanical performance [2, 3].

Over time, right ventricular (R.V.) pacing can induce alterations in the left ventricle and lead to structural remodeling [4]. Furthermore, studies have provided evidence that prolonged right ventricular (R.V.) pacing, particularly when applied in the apical region, is linked to mechanical dyssynchrony. This mechanical dyssynchrony is associated with a decline in left ventricular (LV) systolic function, deterioration in the patient’s functional capacity, and the emergence of psychological issues [5]. In some cases, adverse clinical outcomes of cardiac pacing are documented, such as heart failure, atrial fibrillation, and death [6, 7]. Nevertheless, it should be noted that not all patients undergoing daily clinical practice and receiving R.V. apical pacing will experience these adverse effects [8, 9].

There has been a lack of comprehensive research examining the levels of depression and anxiety in patients both before and after undergoing pacemaker implantation. The existing national and international literature highlights a scarcity of studies focusing on anxiety and depression specifically in individuals with a pacemaker [10, 11], but more research has reported that these two variables are heavily examined in cardiovascular patients [12].

Patients with coronary artery disease may also have depression and anxiety, heart failure [14], and patients who have had an implanted cardioverter defibrillator [15]. It has been suggested that anxiety and depression have a prevalence ranging from 18 to 50% in cardiac patients [16]. Only a few studies that have studied depression and anxiety in pacemaker patients [10, 11] researched these variables only in the time from the implantation and without considering any alterations before and after the pacemaker implantation.

Given the growing elderly population and the subsequent rise in the number of individuals requiring pacemakers, investigating this matter has become crucial. Such studies aim to aid healthcare providers in addressing not only the issue itself, but also identifying those who are susceptible to developing depression and anxiety. Extensive research has demonstrated the profound clinical importance of mitigating anxiety and depression in elderly patient populations. Compelling evidence indicates that promoting mental health and well-being in older adults is associated with enhanced overall wellness, improved quality of life, and reduced healthcare utilization [17, 18].

The main aim of the present study was to determine the predisposed factors with increased levels of depression and anxiety in both before and after pacemaker implantation and determine if any changes in these symptoms occur during 1 year of follow-up after the device implantation.

## Methods

### Ethical considerations

The authors informed patients who had met the mentioned criteria about the study’s aims and shared them only after giving verbal approval. All the patients participated in the study voluntarily. All the questionnaires were anonymous, and consent was obtained to participate in the study. Participants were informed of their right to reject or to finish their participation according to the Helsinki Declaration ethical standards of 1983. The Kafrelsheikh University Hospitals Committee of Medical Research Ethics approved the study. N: MKSU 50-10-12.

### Sample size

Based on that higher level of anxiety prevailed in 40% within the P.P.M. group versus 20% within the control group (Allam et al., 2018), the calculated sample size was 166 divided as 1:1 within case and control group (83 in each group) using Open Epi software, at confidence level 95% and a power of study of 80%. 10% of the sample (eight participants) was added within each group to compensate for potential dropout during follow-up, so control and baseline groups included 91 participants for each during the study. Six patients did not complete the study and were excluded from both groups, so each group included 85 participants who completed the study.

### Design

A prospective cohort study was used to study the effect of the R.V. lead position in dual-chamber pacemaker patients on the patient’s anxiety and depression before, after 6 months, and after 1 year of implantation.

### Study population and procedure

**Fig. 1:**
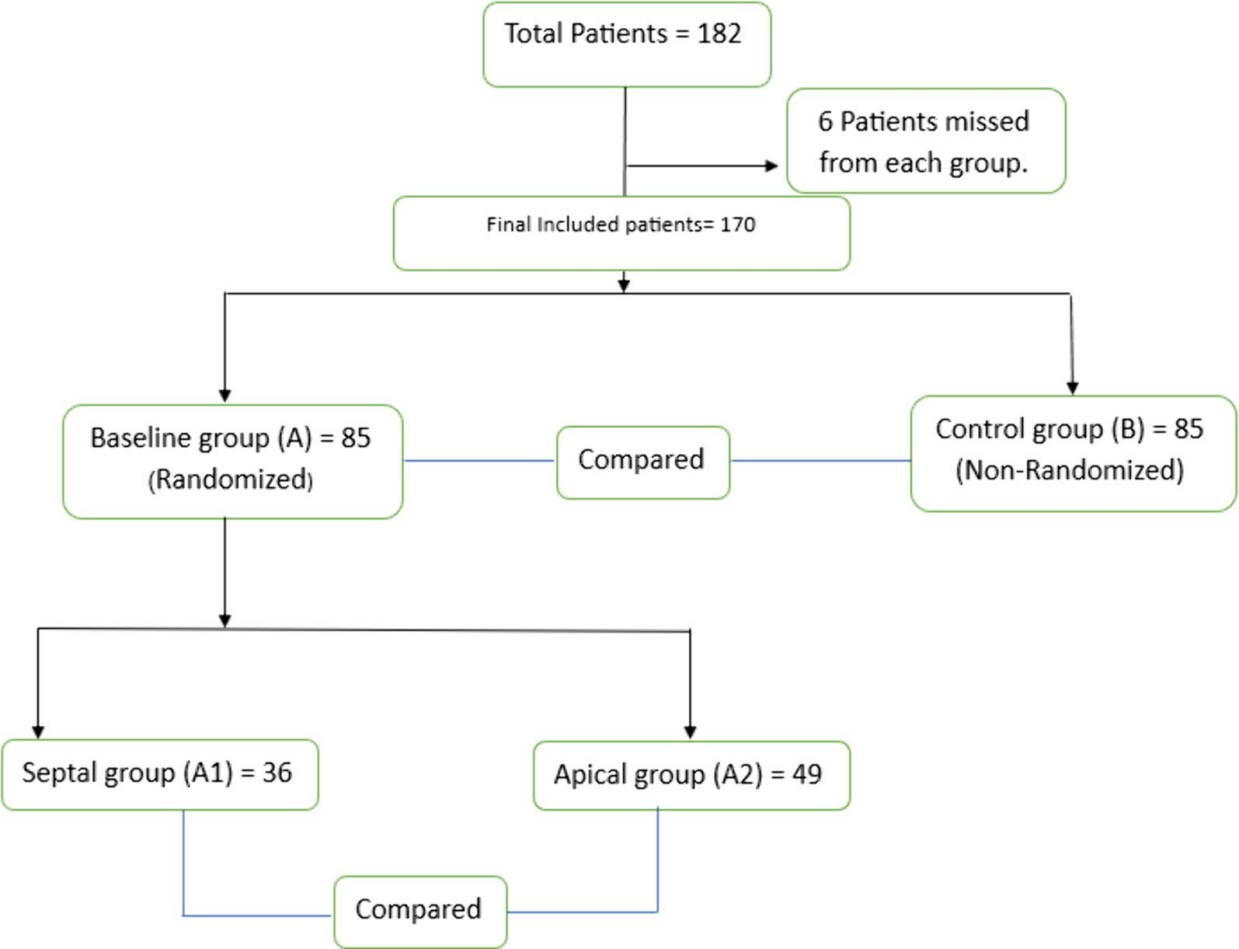
A PRISMA chart illustrating the flow of included participants all over the study

The study included 182 cardiac patients. The patients were enrolled into two main groups: 85 patients before the pacemaker implantation as a baseline (Group A). Those patients were divided according to the site of right ventricular (R.V.) lead position after pacemaker implantation into two subgroups: 36 and 49 patients, respectively, septal (Subgroup A1) and apical (Subgroup A2) lead position and another 85 patients in the same age group to be the control one (Group B) (Fig. 1).

Patients in Group A were recruited to the electrophysiologic study (EPS) clinic before the pacemaker implantation and then randomly listed on a computer system. A specific nurse was responsible for giving a code to each patient in an envelope to ensure that neither the researcher nor the patients knew any information about which group the participant belonged to.

The participants in the septal (A1) and apical (A2) subgroups were drawn from the same patient cohort that comprised the baseline study population. They were included during routine visits in the outpatient clinic of electrophysiology for 6 months and 1 year after implantation; based on the coding system, they had according to (1) the lead position, (2) the way of choice of the lead location during intervention based on the best accessible site during implantation and (3) the accepted programming values during testing the lead location. In the septal group, the pacing is not equivalent to the left septal or attempts at the conduction system, but it just reflects the site of the R.V. leads on the septum. Group B contained cardiac patients in the same age group as the control ones who were not candidates for dual-chamber pacemaker implantation.

The team collected the data in the outpatient cardiology department of Kafrelsheikh University Hospital during the patients’ regular visits for scheduled monitoring and follow-up. Included patients must meet the following criteria: (a) had an indication for dual-chamber pacemaker implantation, (b) the ability to write and read the Arabic language, and (c) patients older than 18 years who were hospitalized for the first time for the implantation procedure. The causes of exclusion of cases were patients with: (a) a history of psychiatric illness (mental, emotional, or behavioral disorders), (b) severe chronic disease, (c) implantable cardioverter defibrillator or biventricular pacing, (d) Patients who did not have stable programming for their devices from the implantation., (e) Patients under 18 years old, (f) Family and social environment problems before and after the pacemaker implant and (g) low socioeconomic standards, low-income patients or any financial problems.

The inclusion criteria of the control group were (a) Stable patients with average levels of anxiety and depression. (b) Patients of the same age group. The criteria of exclusion of control were (a) age less than 18 years, (b) patients who were candidates for pacemaker implantation.

When the selected participants for the study were determined, a clarification of the study aspects was provided, and an approval for their participation was obtained. Participants who agreed to take part in the study received the questionnaires to fill out. The tools were self-administered. Each patient’s data included sociodemographic characteristics (e.g., gender, age, occupation, number of family members), depression and anxiety characteristics (e.g., when the symptoms had started and their score on the scale), other patients’ characteristics, conventional echocardiography and possible pacemaker complications.

The questionnaires took approximately 20 min to complete and occurred when patients came for scheduled monitoring and follow-up visits. So, the team used the Hospital Anxiety and Depression Scale (HADS), which is widely used to determine the levels of experiencing Anxiety and depression.

### Study tools

1. *Sociodemographic Questionnaire*. It was developed to collect data such as age, education, gender, number of family members, occupation, and type of Pacemaker implanted.

2. *Hospital Anxiety and Depression Scale (HADS)* [19] was used to examine patients’ mental health (anxiety and depression). It is formed of a 14-item scale, which assesses patients’ anxiety and depression felt during the previous week. It is divided into two main domains: one for depression (7 items for HADS-D) and another for fear (HADS-A, seven items also). The scores matched to these questions were summed up separately for depression and anxiety; each subscale ranged from zero to twenty-one. A higher score indicated an increase in depression and anxiety. Patients answered every question on a 4-point Likert scale from 0 to 3. The HADS has been widely used for patients with implanted pacemakers [10, 21, 22].

Also, the following configuration has been declared and is widely used in scientific papers: a score of 0–7 refers to no depression or anxiety, a score of 8–10 means moderate levels of depression or anxiety, and a score > 11 reflects increased levels of depression or Anxiety. The HADS scale has been translated, validated, and tested for its reliability in the Egyptian population [20].

### Data from conventional echocardiography

Comprehensive echocardiographic assessments were performed on all study participants at multiple time points, including before pacemaker implantation, immediately after the procedure, prior to hospital discharge, and at 6-month and 1-year follow-ups. These evaluations were conducted using a Philips Epic 7C system equipped with an S5-1 transthoracic transducer and tissue Doppler imaging technology, with subjects positioned in the left lateral decubitus position. The acquired echocardiographic images were digitally stored and subsequently analyzed offline using the dedicated PHILIPS Q-Lab cardiac analysis software, version 15.5. This standardized, state-of-the-art imaging protocol and analysis approach enabled the researchers to obtain a detailed, longitudinal assessment of cardiac structure and function, facilitating a comprehensive characterization of the impact of pacemaker implantation on the study population’s echocardiographic parameters.

Comprehensive echocardiographic evaluation was performed, consisting of standard transthoracic imaging and tissue Doppler techniques. Left ventricular (LV) dimensions and wall thicknesses were measured in the parasternal long-axis view at the level of the mitral valve tips, ensuring perpendicular measurements to the ventricular long axis. Biplane ejection fraction was determined using the modified Simpson’s method, with acquisition of apical four-chamber and two-chamber views. Color flow Doppler was employed to assess cardiac valve function. Right ventricular (RV) dimensions, including basal, mid, and longitudinal axes, as well as RV systolic function parameters, such as tricuspid annular plane systolic excursion (TAPSE) and RV S' wave, were also evaluated. Additionally, pulsed-wave Doppler of the mitral and tricuspid inflow velocities was performed to measure peak early (E) and late (A) diastolic velocities, E-wave deceleration time, and E/A ratio. Tissue Doppler imaging of the lateral and septal mitral annuli, as well as the lateral tricuspid annulus, provided further insights into myocardial diastolic function.

### Pacemaker programming

All patients are programmed in DDDR mode. During the follow-up period, all patients had a programming session to perform the pacing tests, such as sensing, capturing, and impedance as well as revising any documented alarms and checking the battery longevity and the pacing times.

### Screening for complications

A comprehensive screening protocol was followed to detect both acute and chronic complications associated with pacemaker implantation. During and immediately after the procedure, the pacemaker programmer was utilized to select the optimal right ventricular (RV) lead position based on pacing parameters and to identify any abnormal values prior to the patient’s hospital discharge. Additionally, a chest X-ray was performed on all patients just after pacemaker insertion to detect acute complications, such as pneumothorax, hemorrhage/hemothorax, and lead dislocation. One week following the procedure, the surgical wound was assessed for any signs of hematoma, infection, or inflammation. Longer-term, chronic complications, including lead displacement, battery depletion, pacemaker-induced cardiomyopathy (PICM), pacemaker syndrome, Twiddler syndrome, atrial high-rate episodes, and pacemaker malfunction, were monitored using routine echocardiography, the pacemaker programmer, and, if necessary, fluoroscopy to evaluate potential lead displacement.

This comprehensive screening approach, incorporating various diagnostic modalities, enabled the researchers to systematically identify and manage both immediate and delayed complications associated with pacemaker implantation, thereby ensuring the safety and well-being of the study participants.

### Statistical analysis

Data analysis was performed using the software SPSS (Statistical Package for the Social Sciences) version 26. Categorical variables were described using their absolute frequencies and compared using the Chi-square and Fisher exact tests when appropriate. A linear-by-linear association test was used to compare ordinal data between the groups. The Shapiro–Wilk test was used to verify assumptions for use in parametric tests. Normally distributed quantitative variables were described using their means and standard deviations. A paired sample t test (for normally distributed data) was used to compare quantitative data over two-time points. An ANOVA test (for the normally distributed data) was used to compare quantitative data between the two groups. When the difference is significant, the Bonferroni post hoc test was used to detect the difference between each two individual groups. The level of statistical significance was set at *P* < 0.05. A highly significant difference was present if *p* ≤ 0.001.

## Results

The sociodemographic data were presented as follows: The mean age was 61 years in the pacemaker group and 54 years in the control group. The overall patient cohort comprised 60 males and 25 females. After pacemaker insertion, the septal subgroup consisted of 28 males and 8 females, while the apical subgroup included 32 males and 17 females. Lastly, the control group was composed of 52 males and 33 females (Table 1).

**Table 1:** Comparison between the studied groups regarding demographic data

	Septal lead group	Apical lead group	Control group	*χ*^2^	*p*
	*N* = 36 (%)	*N* = 49 (%)	*N* = 85 (%)		
*Gender*					
Male	28 (77.8%)	32 (65.3%)	52 (61.2%)	1.675	0.196
Female	8 (22.2%)	17 (34.7%)	33 (38.8%)		
*Occupation*					
Not working	1 (2.8%)	3 (6.1%)	9 (10.6%)	0.223^¥^	0.637
Unskilled worker	14 (38.9%)	11 (22.4%)	19 (22.4%)		
Skilled worker	8 (22.2%)	10 (20.4%)	18 (21.2%)		
Employee/free trade	3 (8.3%)	8 (16.3%)	11 (12.9%)		
Professional	10 (27.8%)	17 (34.7%)	28 (32.9%)		
*Hypertension*					
Absent	22 (61.1%)	24 (49%)	47 (55.3%)	1.256	0.534
Present	14 (38.9%)	25 (51%)	38 (44.7%)		
*Diabetes*					
Absent	13 (36.1%)	29 (59.2%)	35 (41.2%)	5.622	0.06
Present	23 (63.9%)	20 (40.8%)	50 (58.8%)		
*Ischemia*					
Absent	26 (72.2%)	35 (71.4%)	50 (58.8%)	3.417	0.207
Present	10 (27.8%)	14 (28.6%)	35 (41.2%)		

	Mean ± SD	Mean ± SD	Mean ± SD	*F*	*p*
Age (year)	61.75 ± 11.04	60.55 ± 7.85	54.13 ± 8.11	13.652	< 0.001**
Bonferroni	*P*_a_ > 0.999	*P*_b_ < 0.001**	*P*_c_ < 0.001**		
Weight (kg)	86.25 ± 9.44	85.78 ± 10.42	83.45 ± 9.9	1.394	0.251
Height (cm)	174.28 ± 6.46	175.45 ± 7.0	175.66 ± 11.11	0.292	0.747
BSA (m^2^)	2.02 ± 0.1	2.0 ± 0.11	2.0 ± 0.1	0.874	0.419

*χ*^2^Chi square test ¥ Linear-by-linear association *F* One way ANOVA test **p ≤ 0.001 is statistically highly significant pa difference between septal and apical lead group pb difference between apical lead group and control group pc difference between septal lead and control group

Unskilled workers are widely represented in both cases and control groups, while professional workers were predominately in the sample. B.S.A. was about 2.0 in both cases and control groups. A statistically significant difference is found between the studied groups regarding age. (On doing a post hoc test, the difference is significant between the control group and each other group.) A statistically non-significant difference exists between the studied groups regarding gender, occupation, height, weight, or body surface area. The difference between the studied groups regarding comorbid hypertension, diabetes, or myocardial ischemia was statistically non-significant.

A comparison between the studied groups (with septal and apical leads) regarding demographics, clinical data, and comorbidities was as follows: Over half of the pacemaker patients had an apical lead in their right ventricle (*n* = 49). In contrast, 36 had a septal lead position. Male predominance was evident in apical and septal groups, with an average age of 61 (Table 1**).**

When comparing the studied groups regarding the baseline HADS depression score, there was a statistically non-significant difference (*p* 0.431), while there is a statistically significant correlation between the studied groups regarding HADS depression score after 6 months (*p* 0.013) and 1 year (*p* 0.013). So, the Bonferroni post hoc test was used to identify the statistically significant difference between the control group and the apical lead one. Regarding the HADS anxiety score, there was a statistically non-significant difference between the studied groups at any point regarding baseline (*p* 0.063), after 6 months (*p* 0.054) or even after 1 year of implantation (*p* 0.099) **(**Table 2**).**

**Table 2:** Comparison between the studied groups (septal and apical groups) and the control group regarding HADS depression and anxiety score baseline, after 6 and 12 months

HADS depression score	Septal group	Apical group	Control group	*F*	*p*
	Mean ± SD	Mean ± SD	Mean ± SD		
Baseline	9.5 ± 2.06	9.92 ± 2.2	9.6 ± 2.23	0.462	0.431
After 6 months	9.56 ± 2.93	11.06 ± 3.04	9.65 ± 2.72	4.439	0.013*
Bonferroni	*p*^a^ 0.053	*p*^b^ 0.02*	*p*^c^ > 0.999		
After 1 year	9.47 ± 3.43	11.22 ± 3.85	9.49 ± 3.17	4.477	0.013*
Bonferroni	*p*^a^ 0.063	*p*^b^ 0.016*	*p*^c^ > 0.999		
P1	0.923	0.048*	0.875		
P2	0.789	0.718	0.607		
P3	0.965	0.043*	0.779		
Anxiety score					
Baseline	10.22 ± 2.39	11.2 ± 2.78	10.08 ± 2.79	2.808	0.063
After 6 months	10.25 ± 2.61	11.59 ± 3.05	10.45 ± 3.01	2.978	0.054
After 1 year	9.67 ± 3.38	11.24 ± 3.61	10.32 ± 3.3	2.348	0.099
P1	0.959	0.449	0.282		
P2	0.081	0.513	0.694		
P3	0.388	0.952	0.545		

*p*1 *p* for paired sample *t* test between baseline and after 6 months *p*2 *p* for paired sample *t* test between scale after 6 months and 1 year *p*3 *p* for paired sample *t* test between scale at 1 year and baseline *F One way ANOVA test *p < 0.05 is statistically significant *p*_a_ difference between septal and apical lead group *p*_b_ difference between apical lead group and control group *p*_c_ difference between septal lead and control group

**Fig. 2:**
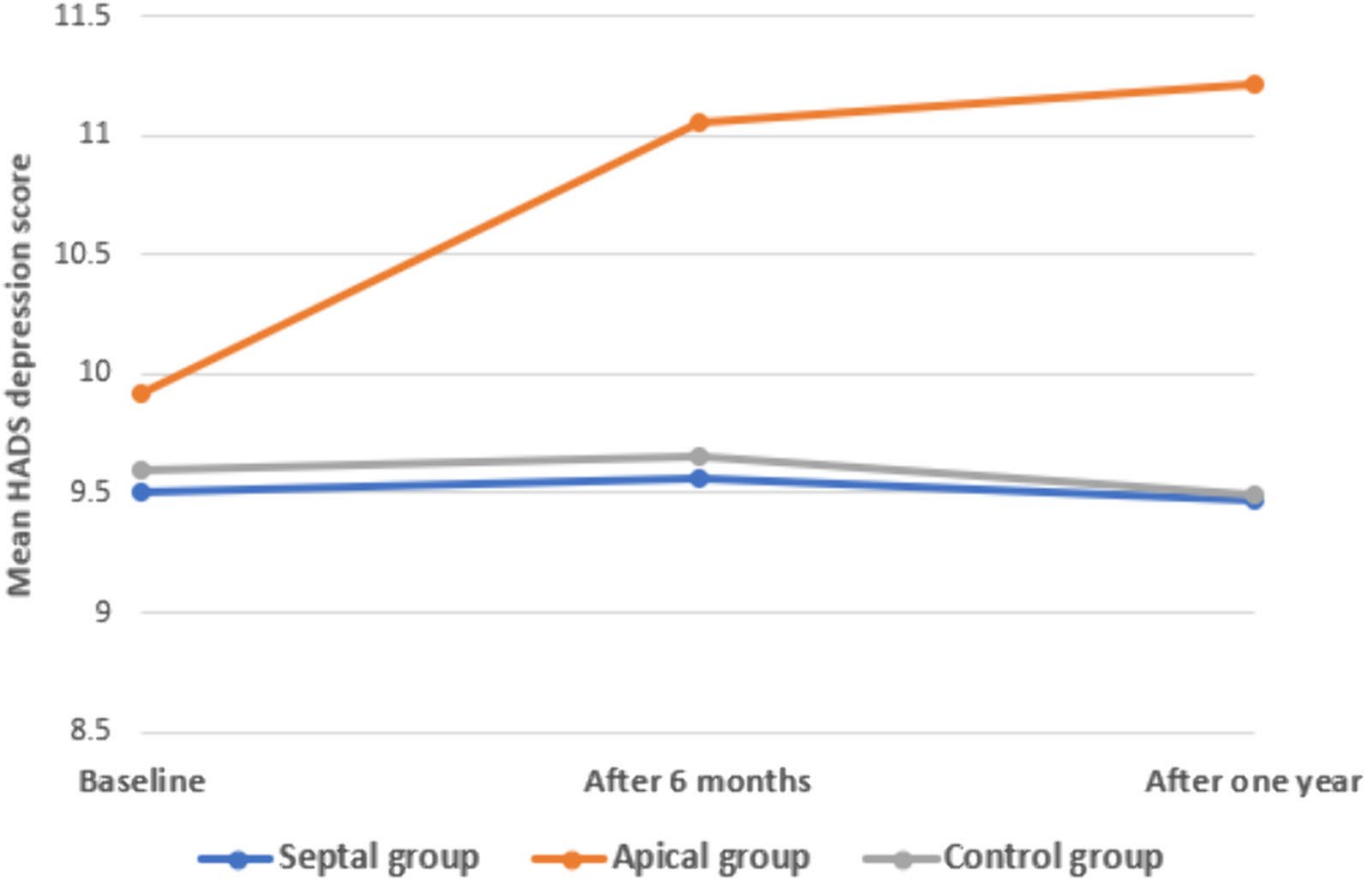
Multiple line graph comparing groups regarding HADS depression score over time

**Fig. 3:**
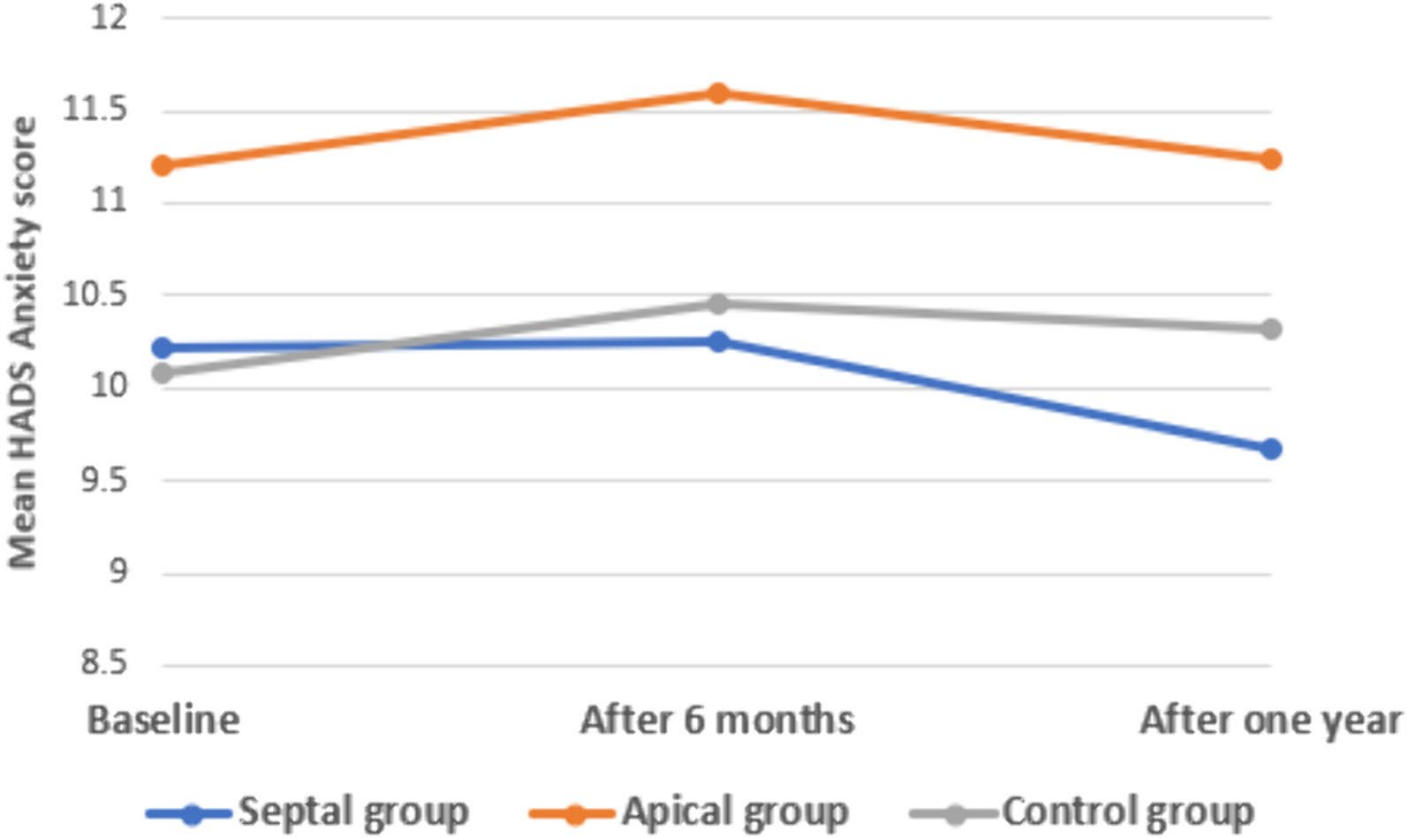
Multiple line graph comparing groups regarding HADS anxiety score over time

Within the control and the septal lead group, there was a non-significant change in the HADS depression score when comparing each two points of time (*p* 0.081). Within the apical lead group, there was a non-statistically significant change in the HADS depression score when comparing every two points of time (*p* 0.513). However, within the apical group, there was a statistically significant increase in HADS depression score after 6 months of implantation as compared to the baseline value before implantation (*p* 0.048). Also, there was a statistically significant increase in depression score after 1 year as compared to the baseline value (*p* 0.043). Within each group, there was a non-significant change in the HADS anxiety score when comparing each two points of time (*p* 0.081) and (*p* 0.513).** (**Figs. 2, 3**).**

According to the linear-by-linear association, there was a statistically non-significant difference between the studied groups regarding the level of anxiety or depression baseline after 6 months or after 1 year. (Table 3**).** All patients depended on the pacemaker during the follow-up periods to maintain their heart rate. The comparison between the studied groups (septal and apical groups) regarding comorbidities showed that there was a statistically non-significant difference between the studied groups regarding the incidence of infection (*p* 0.634), Twiddler syndrome (*p* > 0.999), atrial fibrillation (*p* 0.634), death (*p* > 0.999), or pacemaker malfunction (*p* 0.424). Five patients in the apical group developed cardiomyopathy, which was identified by a drop of E.F. By more than 10% from the baseline, its occurrence was supposed to be related to baseline LVEF% leading to an LVEF of < 50% during the post-implantation period. There was a statistically significant correlation between the HADS anxiety score or depression score after 1 year and the incidence of complications (significantly higher in complicated patients, especially in the apical group) (*p* < 0.001) **(**Table 4**).**

**Table 3:** Comparison between the studied groups regarding levels of anxiety and depression before and after

	Septal lead group	Apical lead group	Control group	*χ*^2^	*p*
	*N* = 36 (%)	*N* = 49 (%)	*N* = 85 (%)		
*Anxiety baseline*					
No	1 (2.8%)	2 (4.1%)	13 (15.3%)	0.224	0.636
Moderate	24 (66.7%)	22 (44.9%)	36 (42.4%)		
Increasing	11 (30.6%)	25 (51%)	36 (42.4%)		
*Anxiety 6 months after*					
No	3 (8.3%)	2 (4.1%)	11 (12.9%)	0.164	0.686
Moderate	18 (50%)	20 (40.8%)	35 (41.2%)		
Increasing	15 (41.7%)	27 (55.1%)	39 (45.9%)		
*Anxiety 1 year after*					
No	8 (22.2%)	5 (10.2%)	13 (15.3%)	3.524	0.06
Moderate	20 (55.6%)	16 (32.7%)	30 (35.3%)		
Increasing	8 (22.2%)	8 (57.1%)	42 (49.4%)		
*Depression baseline*					
No	6 (16.7%)	5 (10.2%)	12 (14.1%)	0	0.99
Moderate	19 (52.8%)	24 (49%)	46 (54.1%)		
Increasing	11 (30.6%)	20 (40.8%)	27 (31.8%)		
*Depression 6 months after*					
No	6 (16.7%)	3 (6.1%)	17 (20%)	0.06	0.807
Moderate	21 (58.3%)	23 (46.9%)	35 (41.2%)		
Increasing	9 (25%)	23 (46.9%)	33 (38.8%)		
*Depression 1 year after*					
No	6 (16.7%)	4 (8.2%)	13 (23.5%)	0.123	0.736
Moderate	23 (63.9%)	20 (40.8%)	30 (36.5%)		
Increasing	7 (19.4%)	25 (51%)	34 (40%)		

*χ*^2^ linear-by-linear association

**Table 4:** Comparison between the studied groups (septal and apical groups) regarding comorbidities

	Septal group	Apical group	χ^2^	*p*
	*N* = 36 (%)	*N* = 49 (%)		
Infection	1 (2.8%)	3 (6.1%)	Fisher	0.634
Heart failure	0 (0%)	5 (10.2%)	Fisher	0.07
Twiddler syndrome	1 (2.8%)	1 (2%)	Fisher	> 0.999
Atrial Fibrillation	1 (2.8%)	3 (6.1%)	Fisher	0.634
Death	0 (0%)	1 (2%)	Fisher	> 0.999
Pacemaker malfunction	1 (2.8%)	0 (0%)	Fisher	0.424

*χ*^2^Chi square test

There is a statistically non-significant difference between the studied groups regarding level of anxiety or depression before pacemaker implantation, after 6 months or even after 1 year of implantation (Table 5).

**Table 5:** Comparison between the studied groups regarding levels of anxiety and depression before and after pacemaker implantation

	Septal lead group	Apical lead group	Control group	*χ*^2^	*p*
	*N* = 36 (%)	*N* = 49 (%)	*N* = 85 (%)		
*Anxiety baseline*					
No	1 (2.8%)	2 (4.1%)	13 (15.3%)	0.224	0.636
Moderate	24 (66.7%)	22 (44.9%)	36 (42.4%)		
Increasing	11 (30.6%)	25 (51%)	36 (42.4%)		
*Anxiety 6 months after*					
No	3 (8.3%)	2 (4.1%)	11 (12.9%)	0.164	0.686
Moderate	18 (50%)	20 (40.8%)	35 (41.2%)		
Increasing	15 (41.7%)	27 (55.1%)	39 (45.9%)		
*Anxiety 1 year after*					
No	8 (22.2%)	5 (10.2%)	13 (15.3%)	3.524	0.06
Moderate	20 (55.6%)	16 (32.7%)	30 (35.3%)		
Increasing	8 (22.2%)	8 (57.1%)	42 (49.4%)		
*Depression baseline*					
No	6 (16.7%)	5 (10.2%)	12 (14.1%)	0	0.99
Moderate	19 (52.8%)	24 (49%)	46 (54.1%)		
Increasing	11 (30.6%)	20 (40.8%)	27 (31.8%)		
*Depression 6 months after*					
No	6 (16.7%)	3 (6.1%)	17 (20%)	0.06	0.807
Moderate	21 (58.3%)	23 (46.9%)	35 (41.2%)		
Increasing	9 (25%)	23 (46.9%)	33 (38.8%)		
*Depression 1 year after*					
No	6 (16.7%)	4 (8.2%)	13 (23.5%)	0.123	0.736
Moderate	23 (63.9%)	20 (40.8%)	30 (36.5%)		
Increasing	7 (19.4%)	25 (51%)	34 (40%)		

*χ*^2^ linear-by-linear association

## Discussion

A statistically significant correlation between the studied groups regarding HADS depression score after 6 months and 1 year (significantly higher in the apical group) was documented in our study and when comparing these results with the baseline group. While within the septal group, there was a non-significant change in HADS depression score over time.

In our present study, most cases were men. Similar notes were made in Germany [1] and the USA [13], with 52.7% and 52.8% of male participants, respectively. According to the results, significant values (*p* 0.036–0.044) of participants had high levels of anxiety after 6 months and 1 year of implantation, respectively. In clinical trials, the importance of these findings was not to compare them with other matched studies but to raise awareness about psychological illness among the patients, who usually needed more care for the technical aspects of the device [8]. Cardiac illness may have a chronic burden, which in turn aggravates depressive episodes as it changes the individual’s feeling of purpose and meaning in life [15, 16].

Anxiety and depression were considered two of the most important factors affecting patients during the period of pre- and post-pacemaker implantation, which in turn affected the patient’s quality of life and behavior during the follow-up period.

In terms of the HADS anxiety score, there was no statistically significant difference observed between the groups under study at any time point, including baseline, 6 months after implantation, or even 1 year after implantation. However, it is important to note that device implantation typically involves a brief hospital stay, and the success of treatment necessitates regular and long-term follow-up to ensure proper device functionality and address any potential practical concerns [21]. As a result, healthcare facilities should promote regular appointments to enhance patient engagement in the therapeutic regimen and uphold self-efficacy behaviors [21, 22].

Many studies discussed the association between Anxiety, depression, and pacemaker implantation according to the type of pacemaker, either single or dual chamber, and either rate-responsive or not. However, our study was unique in measuring anxiety and depression before and after a dual-chamber pacemaker implantation and their affection by the site of proper ventricular lead position either in the RV apex or in the interventricular septum.

The team was on the same line regarding the results and the use of a control group with Allam et al. [23]. This case–control study aimed to investigate the psychological outcome of permanent cardiac pacemakers on pediatric patients and their parents. However, we differed in the patients’ age group as we focused mainly on the adults. The pediatric patients who had undergone permanent cardiac pacemaker implantation had higher levels of depression, anxiety, and PTSD symptoms compared to the control group. The parents of pediatric patients with pacemakers also noticed more elevated levels of anxiety, depression, and PTSD symptoms compared to the control group [23].

Our data agreed with Vellone et al. [24], which aimed to investigate the levels of anxiety and depression in patients before and after pacemaker implantation and the follow-up period, but we had many strength points that made our study more reliable as (1) we had a baseline data for all included patients, (2) the data from all cases was processed in a double-masked randomization process, (3) we focused on the levels of depression and anxiety and (4) the most frequent complications that appeared in the follow-up period.

Highlighting the importance of addressing the psychological well-being of PM patients and providing interventions to manage anxiety and depression may improve their overall quality of life along with our study and with Polikandrioti et al. [25] which investigated the relationship between stress, depression, and fatigue in patients with a permanent pacemaker. They suggested that anxiety and depression might hurt fatigue levels in patients with permanent pacemakers [25].

Also, another remarkable correlation was identified between the studied groups (septal and apical groups) regarding HADS depression score**s,** showing statistically significant differences between them after 6 months and 1 year (significantly higher in the apical group). However, when we made a correlation between the levels of anxiety and depression and the cut-off value for psychiatrist intervention, there were higher levels of depression than anxiety with the apical group more than the septal one but still without a statistically significant difference. So, the patients with apical lead positions need close follow-up as, in the long run, they may need psychiatrist intervention.

Post-implantation complications had a higher level of anxiety and depression as there was a statistically significant difference between the HADS anxiety score or depression score after 1 year and the incidence of complications (significantly higher in complicated patients, especially in the apical group) (*p* < 0.001). Pacemaker-induced cardiomyopathy was identified as more likely with the apical group because of more LV dyssynchrony, as R.V. apical pacing caused heterogeneous LV contraction, which led to deteriorated LV longitudinal contraction detected by echocardiography. Septal pacing could be a better alternative due to less LV dyssynchrony and better longitudinal function than apical pacing, as identified by Inoue K et al. [26].

The pacemaker is widely recognized as a valuable opportunity to enhance patients’ survival rates. Consequently, it necessitates scheduled follow-up visits that include a comprehensive assessment of depression and anxiety, with the provision of psychiatric consultation as necessary.

This study investigated the correlation between anxiety and depression in both the cases and control groups, aiming to identify potential long-term complications and associated factors. The outcomes have the potential to guide healthcare professionals in providing specialized care through the implementation of effective strategies aimed at reducing this burden and aiding patients in managing the challenges associated with their implanted device.

The dropout of some participants during the follow-up period was our main limitation of the current study. Further research will be beneficial in intensifying the association between anxiety/ depression and pacemaker implantation.

## Conclusion

A strong relationship was found between the level of depression and the R.V. site of implantation, as patients with the apical group had higher levels of depression post-implantation. The septal position had less stress and depression on the patient’s well-being than the apical one. Finally, the patients with apical lead positions need close monitoring and follow-up as they may need psychiatrist intervention in the long run.

## Data Availability

Data are available upon request from the corresponding author.

## Abbreviations

RV: Right Ventricle
LV: Left Ventricle
EPS: Electrophysiologic Study
EF: Ejection Fraction
HADS: Hospital Anxiety and Depression Scale
A: Anxiety
D: Depression
PPM: Permanent Pacemaker
PICM: Pacemaker-induced cardiomyopathy
PTSD: Post-traumatic stress disorder
